# Prediction of hepatocellular carcinoma prognosis and immunotherapeutic effects based on tryptophan metabolism-related genes

**DOI:** 10.1186/s12935-022-02730-8

**Published:** 2022-10-10

**Authors:** Chen Xue, Xinyu Gu, Yalei Zhao, Junjun Jia, Qiuxian Zheng, Yuanshuai Su, Zhengyi Bao, Juan Lu, Lanjuan Li

**Affiliations:** 1 State Key Laboratory for Diagnosis and Treatment of Infectious Diseases, National Clinical Research Center for Infectious Diseases, National Medical Center for Infectious Diseases, Collaborative Innovation Center for Diagnosis and Treatment of Infectious Diseases, The First Affiliated Hospital, Zhejiang University School of Medicine, No. 79 Qingchun Road, Shangcheng District, Hangzhou, 310003 Zhejiang China; 2 Division of Hepatobiliary and Pancreatic Surgery, Department of Surgery, The First Affiliated Hospital, Zhejiang University School of Medicine, Hangzhou, Zhejiang China

**Keywords:** HCC, Trp metabolism, Metabolic phenotype, Risk model, Prognosis, Immune escape

## Abstract

**Background:**

L-tryptophan (Trp) metabolism involved in mediating tumour development and immune suppression. However, comprehensive analysis of the role of the Trp metabolism pathway is still a challenge.

**Methods:**

We downloaded Trp metabolism-related genes’ expression data from different public databases, including TCGA, Gene Expression Omnibus (GEO) and Hepatocellular Carcinoma Database (HCCDB). And we identified two metabolic phenotypes using the ConsensusClusterPlus package. Univariate regression analysis and lasso Cox regression analysis were used to establish a risk model. CIBERSORT and Tracking of Indels by DEcomposition (TIDE) analyses were adopted to assess the infiltration abundance of immune cells and tumour immune escape.

**Results:**

We identified two metabolic phenotypes, and patients in Cluster 2 (C2) had a better prognosis than those in Cluster 1 (C1). The distribution of clinical features between the metabolic phenotypes showed that patients in C1 tended to have higher T stage, stage, grade, and death probability than those of patients in C2. Additionally, we screened 739 differentially expressed genes (DEGs) between the C1 and C2. We generated a ten-gene risk model based on the DEGs, and the area under the curve (AUC) values of the risk model for predicting overall survival. Patients in the low-risk subgroup tended to have a significantly longer overall survival than that of those in the high-risk group. Moreover, univariate analysis indicated that the risk model was significantly correlated with overall survival. Multivariate analysis showed that the risk model remained an independent risk factor in hepatocellular carcinoma (p < 0.0001).

**Conclusions:**

We identified two metabolic phenotypes based on genes of the Trp metabolism pathway, and we established a risk model that could be used for predicting prognosis and guiding immunotherapy in patients with hepatocellular carcinoma.

**Supplementary Information:**

The online version contains supplementary material available at 10.1186/s12935-022-02730-8.

## Background

Hepatocellular carcinoma (HCC) ranks as the sixth most common human cancer and accounts for nearly 75–85% of primary liver cancers [1, 2]. Despite the development of therapeutic strategies [3–5], the prognosis of HCC is still poor. The poor prognosis and high death rate of HCC, to a great extent, depend on the limitations of effective treatment for patients with advanced stage HCC [6]. Therefore, assessment of the HCC prognosis risk would greatly benefit the development of available clinical treatments.

Metabolic rewiring in cancer cells is an important hallmark of human cancer [7] that occurs due to the activation of oncogenes, the inhibition of tumour suppressor genes, and/or alterations in signaling pathways [7]. Metabolic alteration modifies the tumour microenvironment and acts key roles in the development of resistance to treatment [8]. L-tryptophan (Trp) is one of the eight essential amino acids that undergoes complex metabolic processes [9]. Trp and its metabolites play an essential role in regulating cellular proliferation and maintenance processes.

Increasing evidence has shown that Trp catabolism participates in immune tolerance through the TRP-kynurenine (KYN) pathway, and it encourages the response to other anticancer drugs [10]. The KYN pathway is the major catabolic pathway for Trp catabolism and begins with the activities of three rate-limiting enzymes, indoleamine 2,3-dioxygenase (IDO1), indoleamine 2,3 dioxygenase 2 (IDO2), and tryptophan-2,3-dioxygenase (TDO2) [11, 12]. IDO1 is the most studied and regulates immune cell function through the KYN pathway, and treatment combining immune checkpoint inhibitors (ICIs) with IDO1 blockade tends to inhibit tumour growth. The IDO1 inhibitor epacadostat has shown potent anti-IDO1 activity through promoting T/natural killer (NK)-cell activation and inhibiting the function of regulatory T cells [13–16]. Considering the strong evidence that TRP metabolism mediates tumour development and immune suppression, a comprehensive analysis of the TRP pathway might improve the development of survival biomarkers and provide potential strategies for the precise treatment of HCC patients.

In this study, we used genes of the Trp metabolism pathway to identify stable metabolic phenotypes by consensus clustering. We also compared the clinical characteristics, pathway characteristics and immune characteristics between the distinct metabolic phenotypes. Finally, we identified differentially expressed genes (DEGs) related to the Trp metabolism phenotype. Furthermore, we established a risk model based on univariate Cox regression analysis and LASSO analysis, which was used for predicting prognosis and developing personalized target therapy in HCC.

## Methods

### Study population and data collection

The available RNA-seq data and follow-up data from patients with HCC were downloaded from The Cancer Genome Atlas Liver Hepatocellular Carcinoma (TCGA-LIHC) dataset, which contains data from 360 HCC tissues and 50 adjacent nontumourous liver tissues. For validation purposes, the gene expression profiles and clinical data from the GSE14520 (including 242 HCC tissues) and GSE76427 (including 115 HCC tissues) cohorts were obtained from the Gene Expression Omnibus (GEO) database. HCCDB18 (including 389 HCC tissues) data were downloaded from the Hepatocellular Carcinoma Database (HCCDB). In this study, we used the TCGA-LIHC dataset as the training set and the GSE14520, GSE76427, and HCCDB18 datasets as independent validation sets.

### Cell culture and Quantitative reverse-transcription PCR (qRT-PCR)

HCC cell lines Hep-G2, Huh-7, Hep-3B, and SK-Hep-1 and normal liver cells LO2 (provide by the China Center for Type Culture Collection) were maintained in DMEM supplemented with 10% fetal bovine serum (FBS) and 1% penicillin/streptomycin (Gibco, USA), which were maintained at 37℃ in a 5% CO2 incubator. The method of qRT-PCR has been described in our previously study [17]. GAPDH was used as control for TPH1. The information of primers sequences in this study were shown in Additional file 5: Table S1.

### Source of tryptophan metabolism-related genes

We extracted the tryptophan metabolism-related genes involved in the tryptophan metabolism pathway "KEGG TRYPTOPHAN METABOLISM" from The Molecular Signatures Database (MSigDB) database (http://software.broadinstitute.org/gsea/msigdb/index.jsp) [18].

### Data preprocessing

We conducted a series of steps to preprocess the TCGA data, including removing the samples without survival time, follow-up data and status. The Ensembl data were converted to gene symbols, and the expression values obtained with multiple Gene Symbols were set at the median value. For the GEO dataset, we downloaded the annotation information of the corresponding chip platform, mapped probes to genes according to the annotation information, and removed probes that matched one probe to multiple genes. When multiple probes matched a gene, the median value was taken as the gene expression value.

### Establishment of risk model

The ConsensusClusterPlus package was used to establish a consistency matrix and cluster HCC patients into distinct subgroups based on the expression data of genes related to tryptophan metabolism [19]. Then, we screened the differentially expressed genes (DEGs) between the two clusters, among which the genes correlated with prognosis were further analyzed (|logfc|> 1 & p < 0.05). Next, we performed lasso Cox regression to compress the core genes that were used for establishing the risk model. The formula used was RiskScore = Σβi × Expi), where i represents the gene expression level, and β is the Cox regression coefficient of the corresponding gene.

### Gene set enrichment analysis (GSEA) and single-sample GSEA (ssGSEA)

To detect the biological signaling pathway, we performed GSEA in distinct clusters built based on tryptophan metabolism-related genes [20]. Here, we performed GSEA against the background of c2.cp.kegg.v7.0.symbols.gmt. Moreover, to observe the relationship between the risk score of patients and their biological functions, we selected the gene expression profiles of each HCC patient to perform ssGSEA based on the R software package. We calculated the scores of each patient in different functions to obtain the ssGSEA enrichment score.

### Cell-type Identification by Estimating Relative Subsets of RNA Transcripts (CIBERSORT)

CIBERSORT is a useful method for characterizing the hematopoietic cell composition of tissues based on RNA transcript profiles [21]. It is usually employed to analyze large-scale gene expression data to develop cellular biomarkers and therapeutic targets [22]. Here, we used the CIBERSORT method to calculate the relative abundance of 22 primary immune cells in distinct subgroups.

### Statistical analysis

Statistical analysis of the data was performed using GraphPad Prism 8 (GraphPad Software Inc., San Diego, CA, USA). Student’s t test of variance was used to calculate the differences between two groups. The survival R package (Version 2.43-3) was adopted to analyze survival rate. p < 0.05 was regarded as statistically significant.

## Results

### Identification of distinct metabolic phenotypes based on Trp metabolism -related genes

**Fig. 1:**
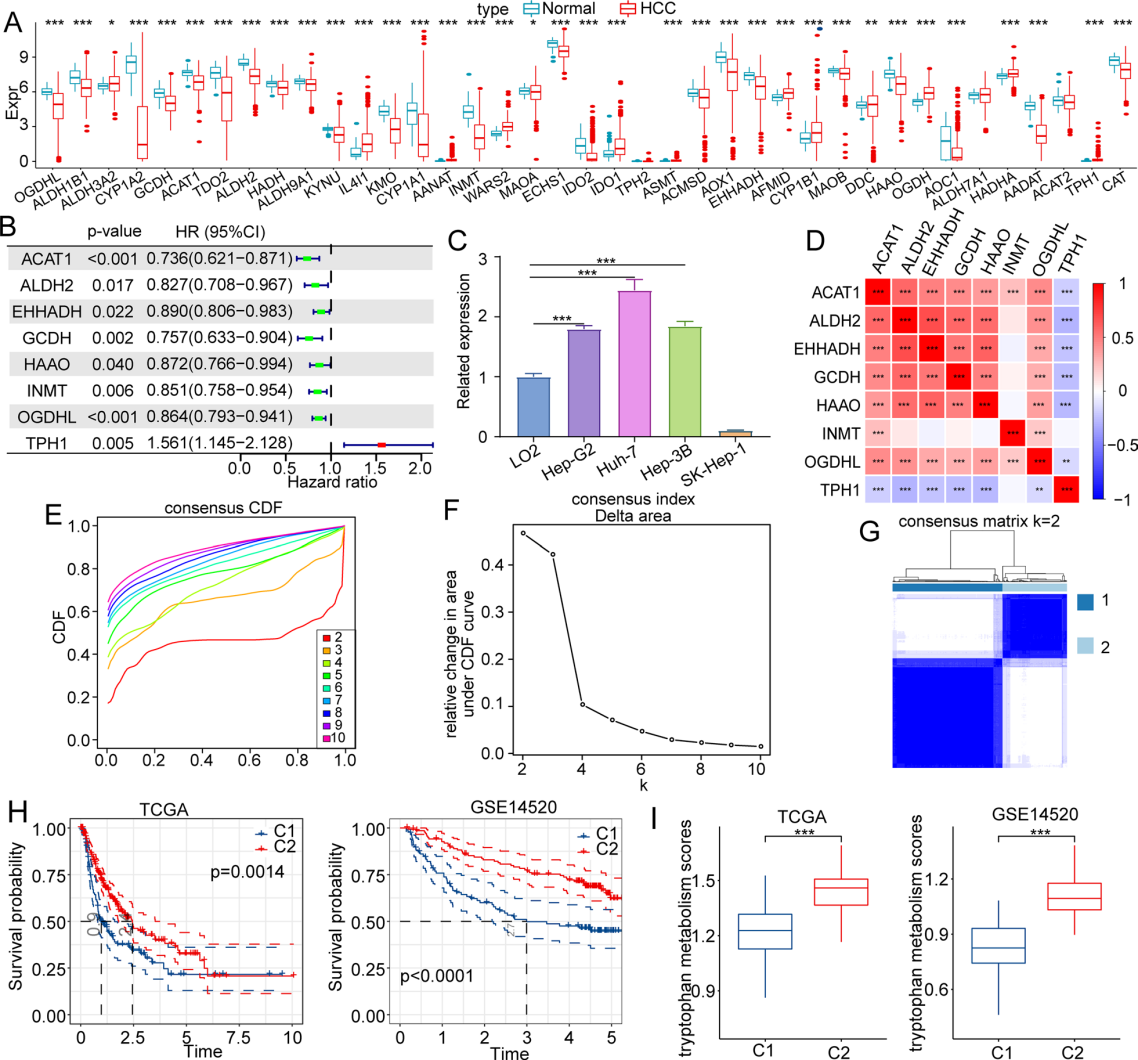
Identification of distinct metabolic phenotypes based on Trp-related genes. **A** The expression profile of Trp metabolism genes in TCGA-LIHC. **B** Forest plot of genes significantly correlated with prognosis. **C** The relative expression level of TPH1 measured by RT-qPCR in HCC cells and normal liver cell line. **D** Heatmap of correlation analysis of prognosis-related genes. E. CDF curve of samples. **F** CDF delta area curve of consensus clustering. **G** The sample clustering heatmap. **H** The survival analysis of the two subtypes in the TCGA-LIHC cohort and GSE14520 cohort. **I** Differences in Trp metabolism scores between C1 and C2 in the TCGA-LIHC cohort and GSE14520 cohort

40 Trp metabolism-related genes were involved in the production of Trp metabolites [23]. Therefore, we compared the mRNA expression levels of Trp metabolism-related genes between HCC tissues and liver tissues in the TCGA dataset. The expression levels of most Trp metabolism-related genes, such as *OGDHL*, *ALDH1B1*, *CYP1A2*, *GCDH*, *ACAT1*, *TDO2*, *ALDH2*, *HADH*, *ALDH9A1*, *KYNU*, *KMO*, *CYP1A1*, *INMT*, *MAOA*, *ECHS1*, *IDO2*, *ACMSD*, *AOX1*, *EHHADH*, *MAOB*, *HAAO*, *AOC1*, *AADAT*, *ACAT2*, and *CAT,* were downregulated in HCC tissues than in paracarcinoma tissues **(**Fig. 1A). To deeply understand the role of Trp metabolism in HCC development and prognosis, we explored the metabolic phenotypes based on Trp metabolism-related genes. First, we conducted a univariate Cox regression analysis to explore the association of the 40 genes with the overall survival of HCC patients collected from the TCGA-LIHC dataset. The expression levels of 8 of 40 genes were significantly correlated with the prognosis of HCC (p < 0.05) (Fig. 1B). As a potential risk factor for *TPH1*, its expression in HCC cells and normal liver cell were tested by qRT-PCR. And results showed that the expression level of *TPH1* was higher in Hep-G2, Huh-7, and Hep-3B compared to LO2 (Fig. 1C). In addition, we observed that there was a significant correlation among the levels of Trp metabolism-related genes using correlation analysis (Fig. 1D). Then, we conducted consistent clustering analysis based on the expression profiles of 8 prognostic genes of Trp metabolism to classify patients. When the number of clusters was selected as 2, we obtained two distinct metabolic phenotypes (Fig. 1E–G). We further analyzed the prognostic characteristics of the two metabolic phenotypes, and we found that patients in C2 had a better prognosis than that of patients in C1. Consistently, we observed similar prognostic features of the two clusters in the GSE14520 dataset (Fig. 1H). In addition, we calculated the "tryptophan metabolism scores" of each patient using the method of ssGSEA in the TCGA and GSE14520 cohort, and we found that the score of C2 with good prognosis was significantly higher than that of C1 (Fig. 1I**)**. These results demonstrated that the Trp metabolism-related clusters demonstrated remarkable differences in the overall survival of HCC patients.

### Clinical features and genetic variation between the two metabolic phenotypes

To further analyze the relationship between different metabolic phenotypes and clinicopathological features in the TCGA-LIHC cohort, we compared the distribution of different clinical features in the two metabolic phenotypes. Patients in C1 tended to have higher T stage, stage, grade, and death probability than those in C2 (Additional file 1: Figure S1). What causes the distinct clinical features between the two metabolic subtypes remained unknown. Therefore, we analyzed differences in genomic alterations between these two metabolic phenotypes. To this end, we obtained information on the molecular features of the TCGA-LIHC dataset from a previous pancancer study [24]. In this published study, the authors classified HCC patients into 5 immune subtypes, of which subtypes 3 and 6 had the best prognosis, and immune subtype 1 had the worst prognosis (Additional file 2: Figure S2A). Here, we found that C1 showed a higher aneuploidy score, more homologous recombination defects, and different fractions than those of C2 (Additional file 2: Figure S2B). Furthermore, we found that immune subtype 3 among the immune molecular subtypes was more prevalent in the patients of the C2 metabolic subtype who had good prognoses, while immune subtype 1 was more prevalent in the patients of the C2 subtype who had poor prognoses (Additional file 2: Figure S2C). Furthermore, we explored the differences in gene mutations between different metabolic phenotypes, and the top 10 genes with significant differences are shown in Additional file 2: Figure S2D. We found that the mutation frequencies of various genes, such as those in *TP53*, *TTN* and *MUC16*, were significantly different between the two metabolic phenotypes.

### The difference in immune cell infiltration characteristics and immunotherapy/chemotherapy response between the two clusters

**Fig. 2:**
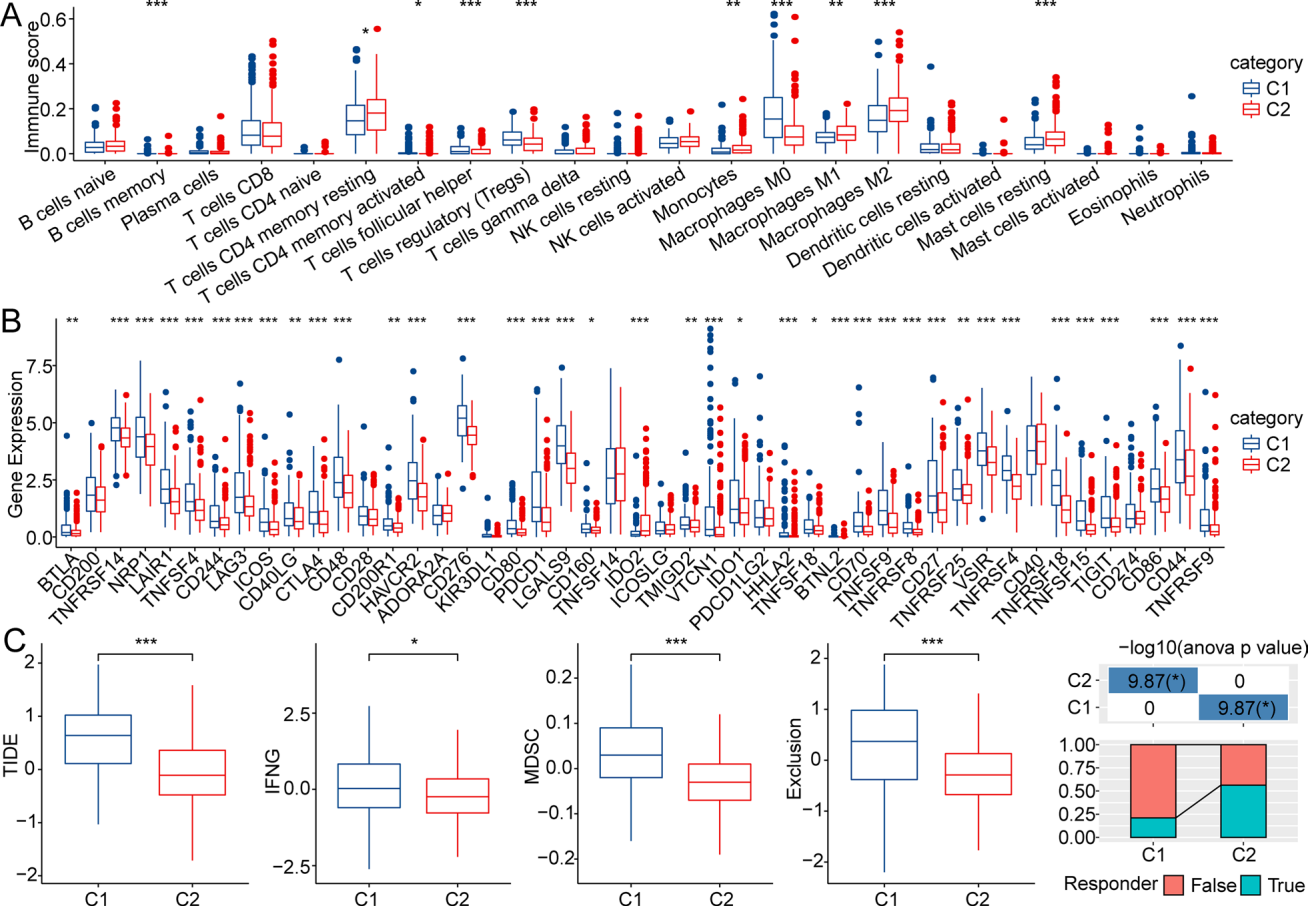
The differences in the immune cell infiltration characteristics and immunotherapy/chemotherapy response between C1 and C2. **A** Different infiltrating levels of 22 immune cells between the two molecular subtypes. **B** The differential expression of ICI genes between C1 and C2. **C** Differences in TIDE scores between C1 and C2

The immune microenvironment plays a key role in the development of HCC and the response to immune checkpoint blockers (ICBs) [8, 25, 26]. Therefore, we elucidated the immune microenvironment of patients with different metabolic phenotypes. In the TCGA-LIHC cohort, we assessed the extent of immune cell infiltration in patients by calculating the expression levels of genes in immune cells via CIBERSORT. Many types of immune cells, including CD4 + memory-activated T cells, monocytes, and M1 macrophages, were more abundant in C2 than in C1 (Fig. 2A). The abundance of infiltrating immunosuppressive cells, including that of regulator T cells, for example, was higher in C1 than in C2 (Fig. 2A). Moreover, we detected whether there were differences in the response to immunotherapy between different metabolic phenotypes. First, we explored the immune checkpoint genes expression levels, and the results showed that the expression levels of most of the immune checkpoint genes, such as *CTLA4*, *IDO1*, *TNFSF18*, *TNFSF4*, *TNFSF9*, and *NRP1*, were higher in C1 than in C2 (Fig. 2B). Then, we compared the differences in the potential effects of immunotherapy between different metabolic phenotypes by Tracking of Indels by Decomposition (TIDE) software. The higher the TIDE prediction score was, the higher the possibility of immune escape and the lower the possibility of patients benefiting from immune therapy (Fig. 2C). As expected, the TIDE score in C1 was higher than that in C2 in the TCGA cohort, suggesting that C1 patients have a higher possibility of exhibiting immune escape. All the results suggested that the metabolic subtypes might be used for assessing the immune microenvironment and predicting the response to immunotherapy for patients with HCC.

### Pathway enrichment analysis of the two metabolic subtypes

To further explore the difference in the underlying regulatory mechanisms of C1 and C2, we employed GSEA in the TCGA cohort. Results showed that some pathways, including the Notch signaling pathway and the pathway related to pathogenic Escherichia coli infection, were enriched in C1, while more pathways, such as tryptophan metabolism, peroxisome, propanoate metabolism, retinol metabolism, valine, leucine and isoleucine degradation pathways, were enriched in C2 (Additional file 3: Figure S3A). We also analyzed the differences in the enrichment of 10 oncogenic pathways (WNT, TP53, RAS, TGF-beta, PI3K, NRF1, NOTCH, MYC, HIPPO, and cell cycle) in the two metabolic subtypes in a previous study [27]. We observed significant differences in most types of pathways except TGF-beta and MYC pathways (Additional file 3: Figure S3B).

### Establishment of a novel risk model for predicting the prognosis of patients with HCC

**Fig. 3:**
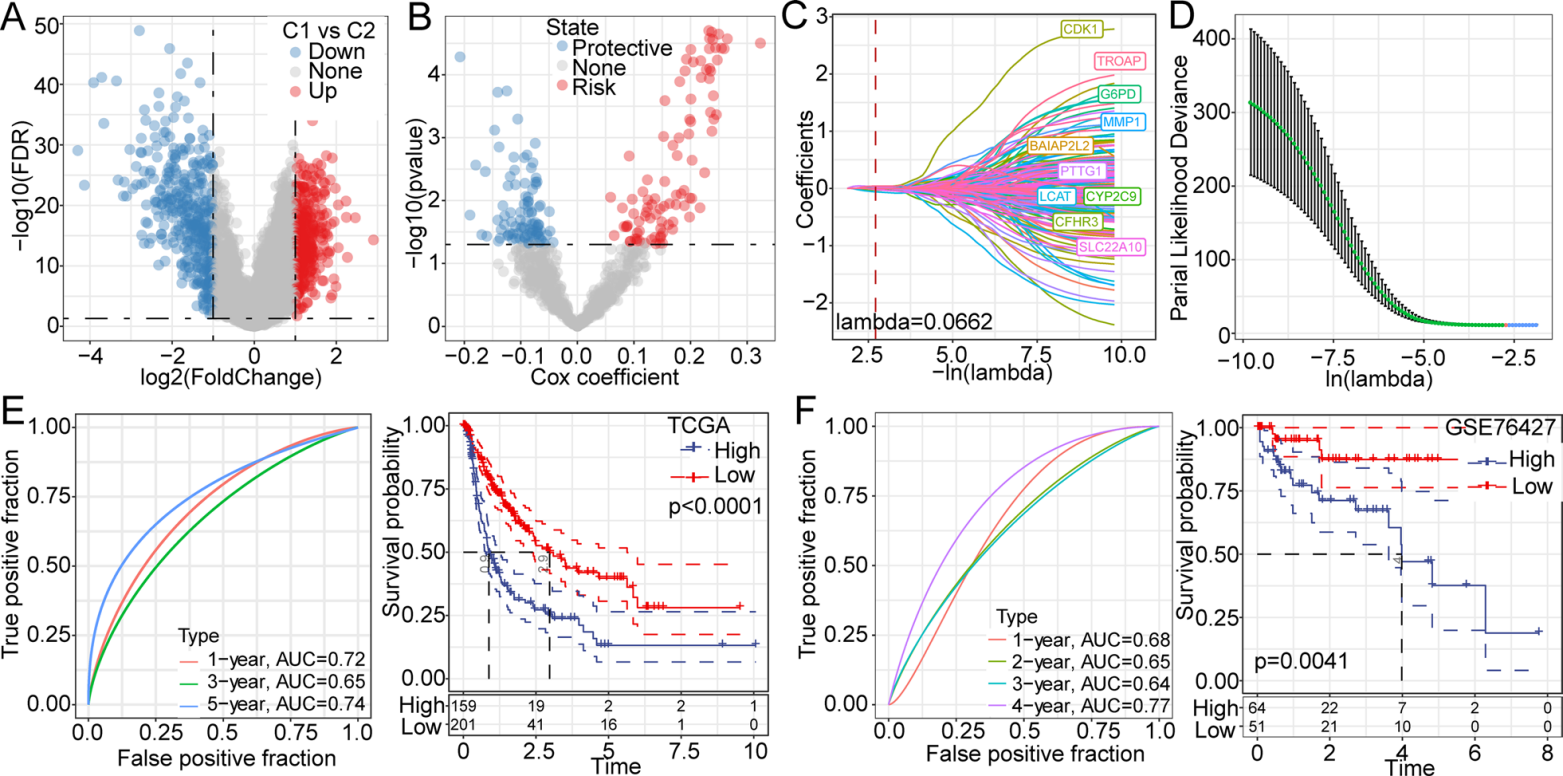
Establishment of a novel risk model based on the DEGs between C1 and C2. **A** Volcano plot of DEGs. **B** The differentially expressed genes were analyzed by univariate regression. **C** The trajectory of each independent variable with lambda. **D** Confidence interval under lambda. ROC curve and survival analysis were used to construct a risk model in the TCGA-LIHC dataset (**E**) in the GSE76427 dataset (**F**)

In the above analysis, we identified two distinct metabolic subtypes based on the genes associated with Trp metabolism. Next, we used the limma package to calculate the differentially expressed genes (DEGs) between C1 and C2 (FDR < 0.05 and |log2FC|> 1). Finally, we screened 739 DEGs between the clusters. All the DEGs are shown in the volcano plot (Fig. 3A). The univariate Cox regression model identified 189 genes that were markedly associated with prognosis (Fig. 3B). Among 189 genes, 95 genes were risk genes. Then, lasso Cox analysis was adopted to further compress the number of key genes (Fig. 3C). When lambda = 0.066, the model reaches the optimum (Fig. 3D). Therefore, we chose the 10 identified genes (*cyclin-dependent kinases* (*CDK1*), *TROAP, G6PD, MMP1, BAIAP2L2, PTTG1, LCAT, CYP2C9, CFHR3, and SLC22A10)* for which lambda = 0.066 for further study.

Based on the ten-gene model, the AUCs for 1-, 3-, and 5-year overall survival were 0.72, 0.65, and 0.74, respectively (Fig. 3E). Additionally, we found that patients in the group with low risk scores tended to have significantly longer overall survival than those in the group with high risk scores based on TCGA cohort (Fig. 3E). Furthermore, similar AUCs and prognostic differences were observed in the GSE76427 cohort (Fig. 3F).

### Performance of the risk model in patients with different clinicopathological features

To examine the relationship between the risk score and clinical features of HCC, we analyzed the difference in risk score between patients with different TNM grades and stages. As the clinical grade increased, the risk score increased, which indicates that patients with higher clinical grades had higher risk scores (Additional file 4: Figure S4A). Additionally, patients in the high-risk-score groups had a higher T stage, stage, and grade than those in the low-risk group (Additional file 4: Figure S4B). This evidence demonstrated that the risk score could function as a biomarker for prognostic prediction and may provide clues for developing precise treatment strategies.

### Immune cell infiltration characteristics in distinct risk score subgroups

**Fig. 4:**
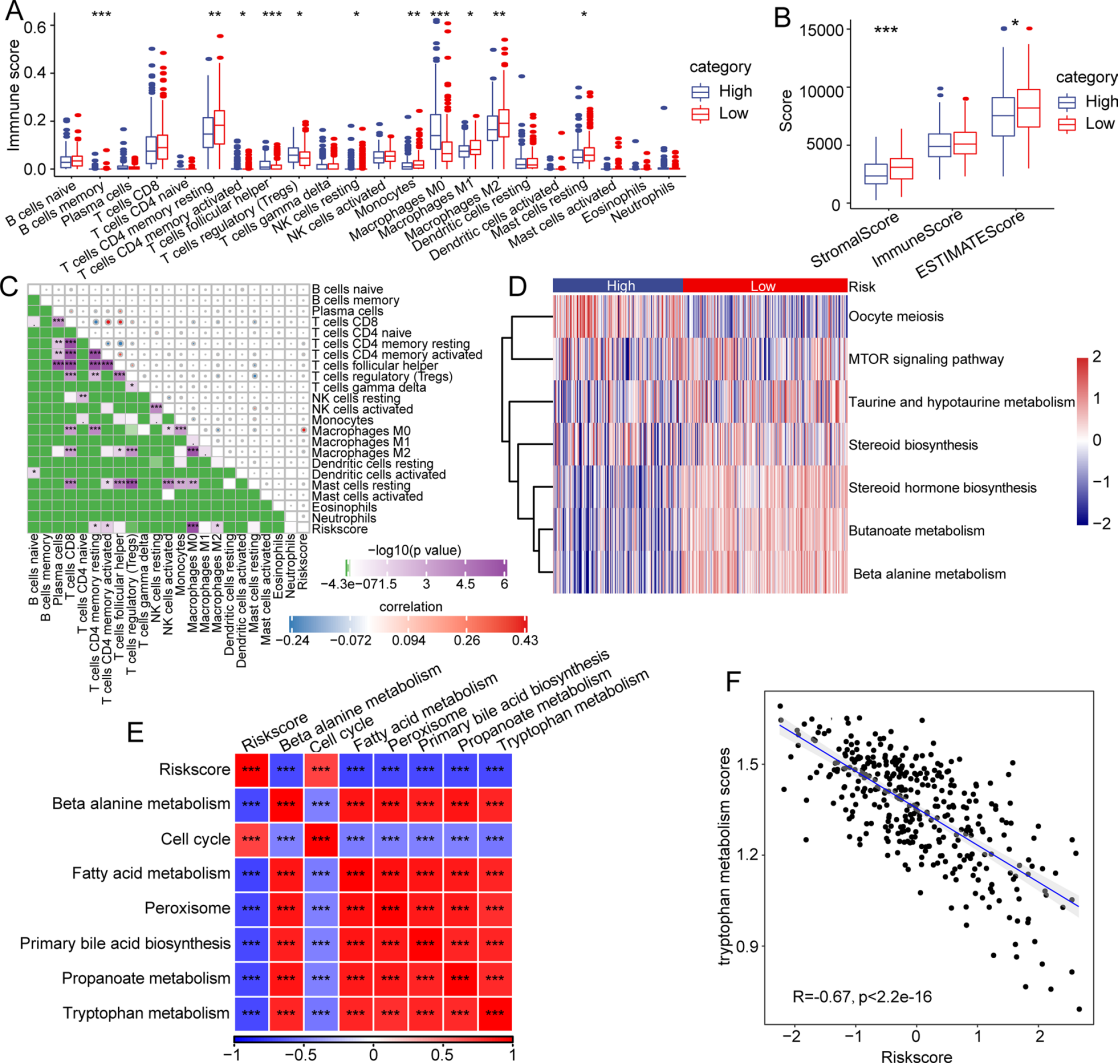
Immune cell infiltration characteristics in distinct risk subgroups. **A** Boxplot of differences in the infiltrating abundance of 22 immune cells between different risk subgroups. **B** Boxplots of differences in immune scores calculated between the risk subgroups by the ESTIMATE method. **C** Correlation between 22 immune cell components and risk score. **D** Heatmap of enrichment scores of pathways. **E** Correlation analysis between risk score and the pathways (R > 0.7). **F** The correlation between the risk score and the tryptophan metabolism pathway

To further clarify the characters in different scores, we compared the abundances of infiltrating immune cells in the groups (Fig. 4A). We observed significant differences in the abundances of many immune cells, such as memory B cells, resting CD4 + T cells, M0 monocytes, M1 monocytes, M2 monocytes, and resting mast cells, between the risk subgroups. We also performed ESTIMATE to explore the immune cell infiltration levels (Fig. 4B). And we found that the risk score was markedly related with the abundances of CD4 + T cells and macrophages (Fig. 4C**)**. We performed ssGSEA based on TCGA-LIHC cohort, and Oocyte meiosis was mainly enriched in the high-risk score subgroup, while steroid hormone biosynthesis, butanoate metabolism and beta alanine metabolism were mainly enriched in the low-risk score subgroup **(**Fig. 4D). Additionally, the correlation between these biological functions and the risk score was further assessed (Fig. 4E). The risk score was positively associated with the cell cycle but negatively correlated with beta alanine metabolism, fatty acid metabolism, peroxisome, primary bile acid biosynthesis, propanoate metabolism and tryptophan metabolism(Fig. 4 E). Notably, we found a remarkably negative correlation between the risk score and tryptophan metabolism ssGSEA scores. The risk score was closely associated with tryptophan metabolic pathways and could be used to indirectly assess the immune microenvironment (Fig. 4F).

### The risk model has excellent predictive power for immunotherapy and chemotherapy for HCC

**Fig. 5:**
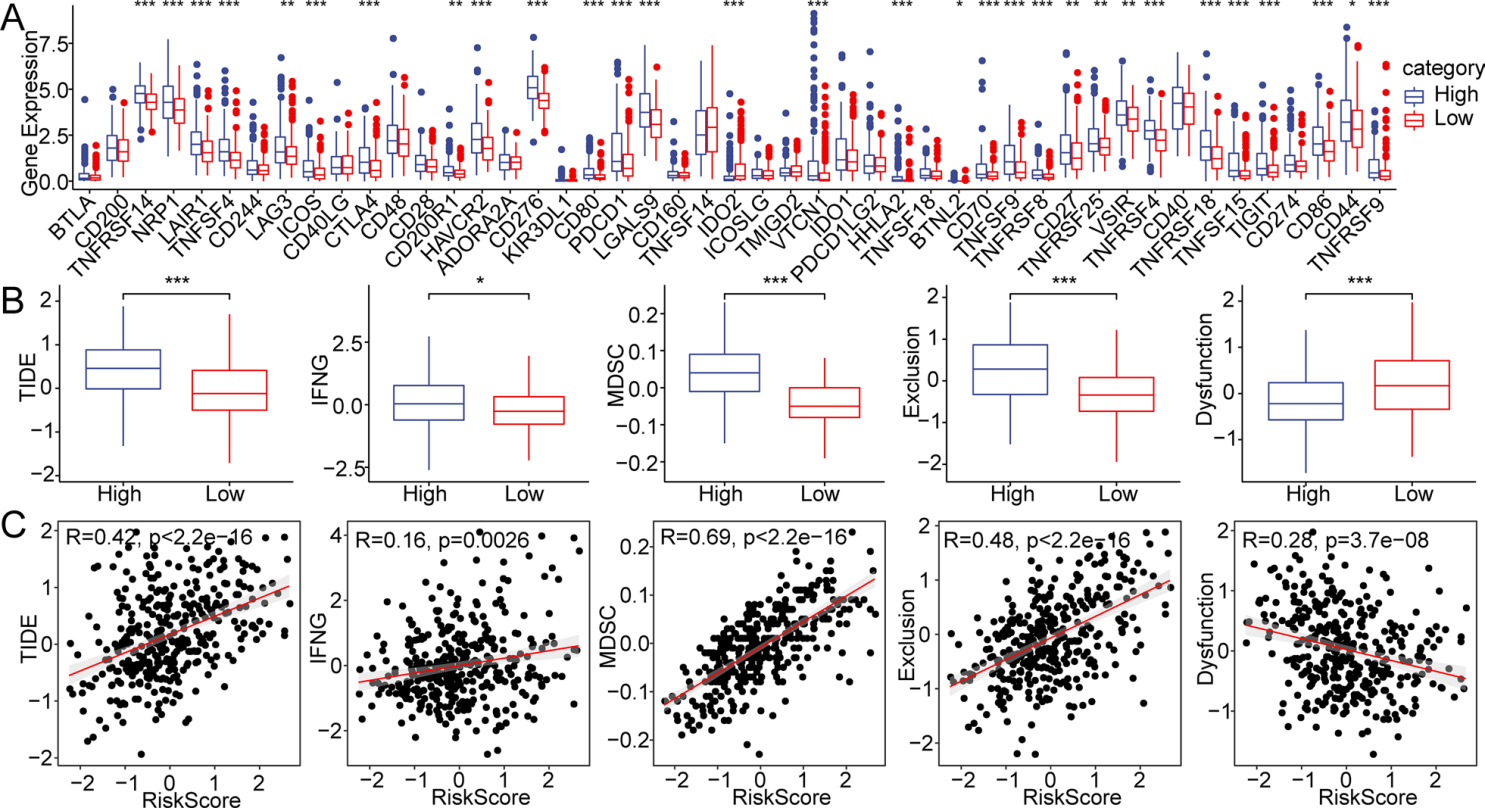
The risk model has excellent predictive power for immunotherapy and chemotherapy for HCC. **A** Differentially expressed immune checkpoint genes between different risk subgroups. **B** Differences in TIDE scores between different risk subgroups. **C** Correlation between risk score and TIDE scores

Immunotherapy for HCC is promising but particularly challenging due to the complex immune microenvironment and lack of reliable biomarkers for immunotherapy [28]. Here, we calculated the expression levels of immune checkpoint genes between risk subgroups. The results showed that the expression levels of various genes, such as *TNFRSF14, NRP1, LAIR1, TNFSF4, CD276, CD80, CD44*, and *CD86*, were higher in the high-risk score subgroups (Fig. 5A). Then, we analyzed the differences in the effects of immunotherapy between different risk score subgroups using TIDE software. The high-risk subgroup had a higher TIDE score, suggesting that this group had a higher possibility of immune escape (Fig. 5B). Further research showed that there was a significant correlation between the risk score and the TIDE, interferon gamma (IFN-γ), myeloid-derived suppressor cell (MDSC), and exclusion scores (Fig. 5C).

### The risk score served as an independent biomarker for predicting the prognosis of patients with HCC

**Fig. 6:**
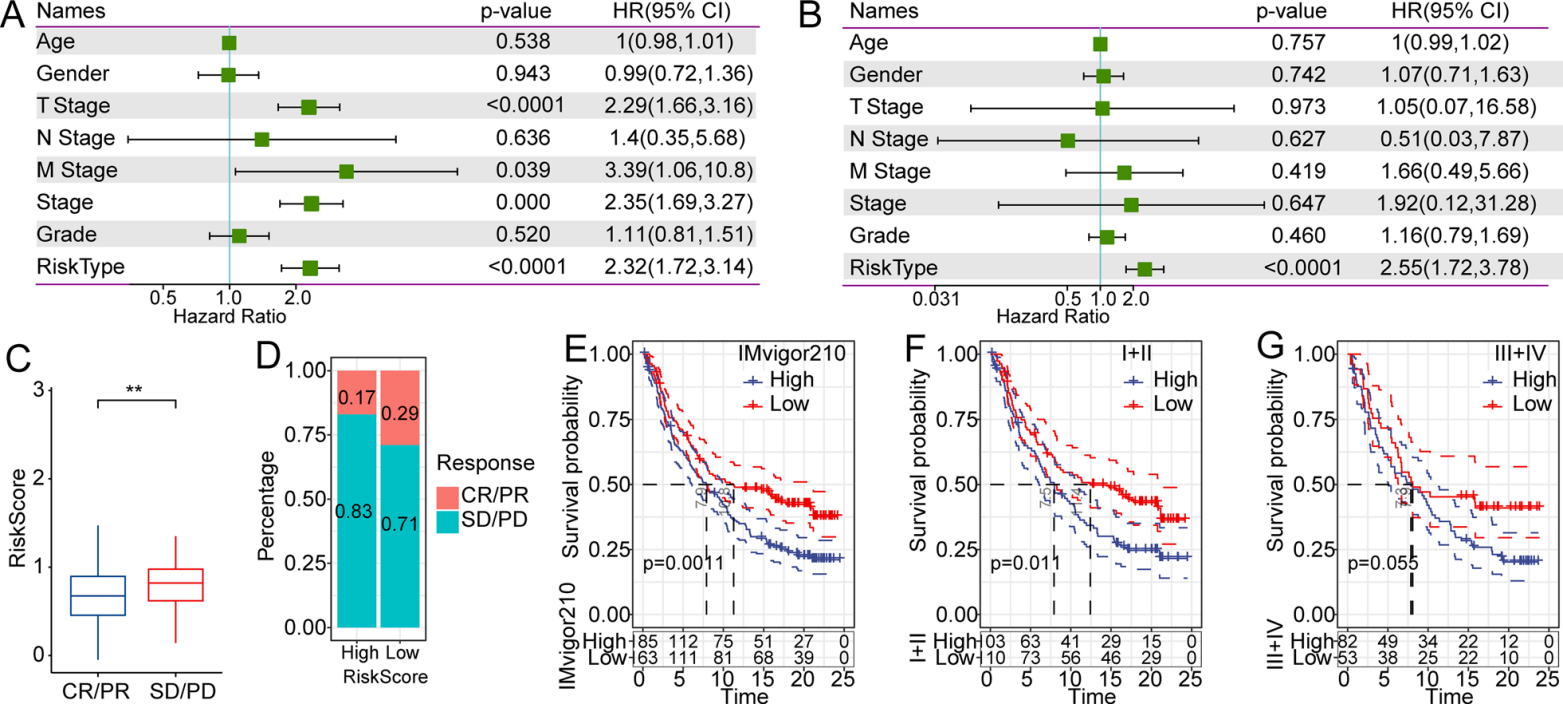
Clinical application of risk models for predicting prognosis and response to immunotherapeutic effect. **A** Univariate Cox analysis of clinical characteristics and RiskType based on TCGA database. **B** Multivariate Cox analysis of clinical characteristics and RiskType. **C** In the IMvigor210 cohort, SD/PD patients had higher risk scores than other types of responders. **D** The percentage statistics showed that the treatment effect was significantly better in the low-risk group than in the high-risk group. **E** Prognostic difference in risk subgroups in the whole TCGA-LIHC cohort. Prognostic difference in early-stage patients in the IMvigor210 cohort. Prognostic difference between different risk groups of early-stage patients (**F**) and late-stage patients in the IMvigor210 cohort (**G**)

HCC patients with clinical information were selected for further analysis. Univariate analysis indicated that T stage, M stage, pathological stage and risk model were significantly related with overall survival (Fig. 6A). After the multivariate analysis, only the risk model remained an independent risk factor associated with prognosis (Fig. 6B). Our findings indicated that the risk model had excellent predictive efficiency for prognostic prediction in HCC.

To observe immunotherapy in the different risk score, we assessed the ability of the risk model to explore the response to ICIs in HCC patients. In the IMvigor210 cohort, patients were varied into complete response (CR), partial response (PR), stable disease (SD), and progressive disease (PD). SD/PD patients had higher risk scores than those of other types of responders (Fig. 6C). The percentage statistics indicated that the treatment effect was better in the low-risk score group (Fig. 6D). We analyzed the survival difference in all samples in IMvigor210, and we observed that patients with a higher risk score were related with a poorer survival rate (Fig. 6E**)**. In addition, there was a significant survival difference in early stage and advanced stage, respectively (Fig. 6F and G). All the results suggest that the ten-gene model could be used for the assessment of immunotherapy efficacy and for guiding therapeutic strategies for patients with HCC.

## Discussion

In this study, we combined a local sample bank and a public database to explore the expression of 40 Trp metabolism -related genes in HCC. We observed that the levels of most Trp metabolism -related genes were lower in HCC tissues than in normal tissues, suggesting that the Trp metabolism pathway could play key roles in HCC development.

Mounting studies have confirmed that Trp, and especially the KYN pathway, mediates tumour tolerance, and accumulating levels of tryptophan catabolites in HCC cells increases the malignant properties of cancer cells, suppresses the antitumour immune response and promotes tumour cell immune invasion [12, 29, 30]. Many studies have found that reduced Trp levels contribute to the suppression of tumour growth in adult T-cell leukemia [31], lung cancer [32], colorectal cancer [33] and glioma [34]. There is evidence that IDO1 suppresses T-cell responses by facilitating Treg cell activation and/or differentiation [35–37]. Fallarino et al. reported that IDO has highly versatile regulator functions driven by distinct cytokines; thus, the IFN-γ-IDO axis is involved in regulating the function of regulatory T cells [38]. However, HCC is a highly heterogeneous cancer, and the tumour microenvironment includes tumour cells and various types of immune cells. It is not easy to thoroughly understand the metabolism of tumour cells and the functional status of immune cells. Here, we focused on the whole genes of Trp metabolism in HCC based on the local sample cohort and TCGA-LIHC cohort and identified two distinct metabolic phenotypes. Importantly, we comprehensively analyzed the expression of the ICI gene targets and the infiltrating abundance of various types of immune cells in the tumour microenvironment between the two metabolic phenotypes. The results demonstrated the clinical utility of the distinct phenotypes in predicting treatment benefits in HCC.

Biomarkers can serve as tools in prognostic prediction to assess treatment response, surveil tumour recurrence, and guide therapeutic approaches [39]. In addition, we established a new risk model with high performance for predicting the prognosis and response to immunotherapy in HCC. This model includes ten genes with multiple functions in cancer. Cyclin-dependent kinase 1 (CDK1) is a protein kinase that plays an important role in the cell cycle. Trophinin-associated protein (TROAP) has been reported to be highly expressed and promotes tumour progression in HCC [40], prostate cancer [41], breast cancer [42], glioma [43], gastric cancer [44], and clear cell renal cell carcinoma [45]. BAIAP2L2 [46, 47], pituitary tumour-transforming gene 1 (PTTG1) [48, 49] and complement factor H-related 3 (CFHR3) [50] have also been reported to be overexpressed in many types of human cancer and to promote tumour cell angiogenesis, migration and invasion. However, the advantage of our study was that the expression levels of these gene were assessed in large samples of HCC tissues. In addition, the ten-gene-based risk model had excellent predictive performance for the prognosis of patients with HCC. Moreover, because there are few published studies combining TRP metabolism and the immune microenvironment, we examined the relationship of the risk score and tumour immune escape. The evidence demonstrated the clinical utility of the ten-gene model in predicting the benefit of immune treatment in HCC.

In this study, the risk model was validated in another dataset and showed accurate prediction of survival. Although this model performs well in predicting HCC prognosis, there are several limitations in the present study. First, the model was built based on a published database, and caution should be taken when extending our research findings to local patients. Second, the function and regulatory mechanisms of the ten genes in vitro and vivo need further investigation. Third, further prospective studies are necessary for the validation of the clinical implications of the risk model.

## Conclusions

Overall, we identified two metabolic phenotypes with distinct characteristics of immune cell infiltration based on the Trp metabolism-related genes. Furthermore, we established a ten-gene risk model for the prediction of prognosis and immunotherapeutic response in HCC.

## Supplementary Information

**Additional file 1: Figure S1.** Distribution of clinical information between the C1 and C2 based on TCGA database.

**Additional file 2: Figure S2.** Genomic alterations of metabolic subtypes in the TCGA-LIHC cohort.

**Additional file 3: Figure S3.** Enrichment analysis of metabolic subtypes.

**Additional file 4: Figure S4.** Characters in different clinicopathological features.

**Additional file 5: Table S1.** The information of primers sequences for qRT-PCR assay.

## Data Availability

The datasets used and/or analysed during the current study are available from the corresponding author upon reasonable request.

## Abbreviations

Trp: L-tryptophan
GEO: Gene Expression Omnibus
HCCDB: Hepatocellular Carcinoma Database
TIDE: Tracking of Indels by DEcomposition
C2: Cluster 2
C1: Cluster 1
DEGs: Differentially expressed genes
AUC: Area under the curve
HCC: Hepatocellular carcinoma
KYN: TRP-kynurenine
IDO1: Indoleamine 23-dioxygenase
IDO2: Indoleamine 23 dioxygenase 2
TDO2: Tryptophan-23-dioxygenase
ICIs: Immune checkpoint inhibitors
TCGA-LIHC: The Cancer Genome Atlas Liver Hepatocellular Carcinoma
MSigDB: The Molecular Signatures Database
GSEA: Gene set enrichment analysis
ssGSEA: Single-sample Gene set enrichment analysis
CIBERSORT: Cell-type Identification by Estimating Relative Subsets of RNA Transcripts
ICBs: Immune checkpoint blockers
IFN-γ: Interferon gamma
MDSC: Myeloid-derived suppressor cell
CR: Complete response
PR: Partial response
SD: Stable disease
PD: Progressive disease
CDK1: Cyclin-dependent kinase 1
TROAP: Trophinin-associated protein
PTTG1: Pituitary tumour-transforming gene 1
CFHR3: Complement factor H-related 3
