# Supplementary figures and images for: Prediction of hepatocellular carcinoma prognosis and immunotherapeutic effects based on tryptophan metabolism-related genes
Source: Cancer Cell Int. 2022 Oct 10;22:308. doi: 10.1186/s12935-022-02730-8 (PMC9552452; doi:10.1186/s12935-022-02730-8)

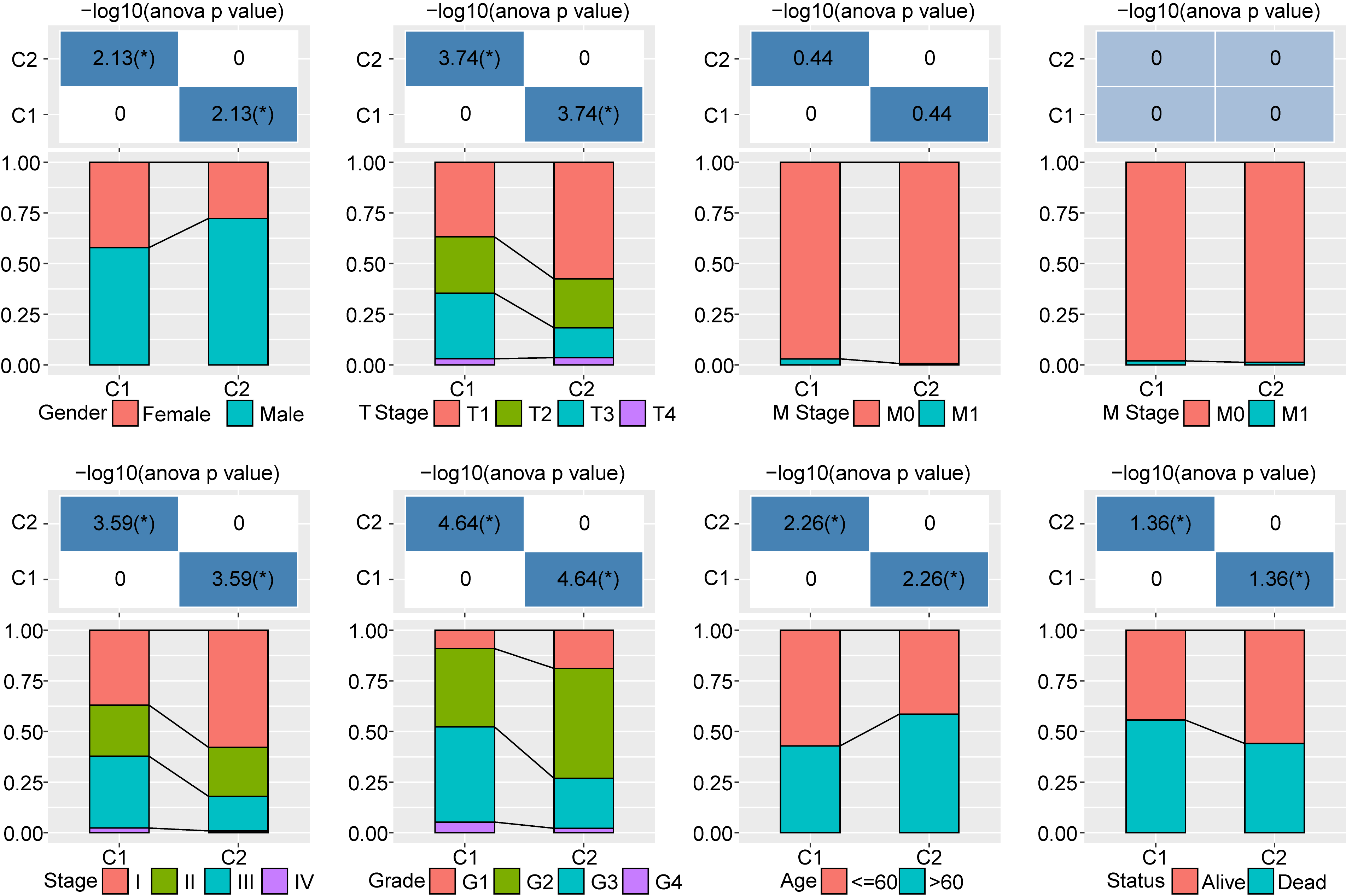

Supplement: Supplementary file 1 — Additional file 1: Figure S1. Distribution of clinical information between the C1 and C2 based on TCGA database. [file 12935_2022_2730_MOESM1_ESM.tif]

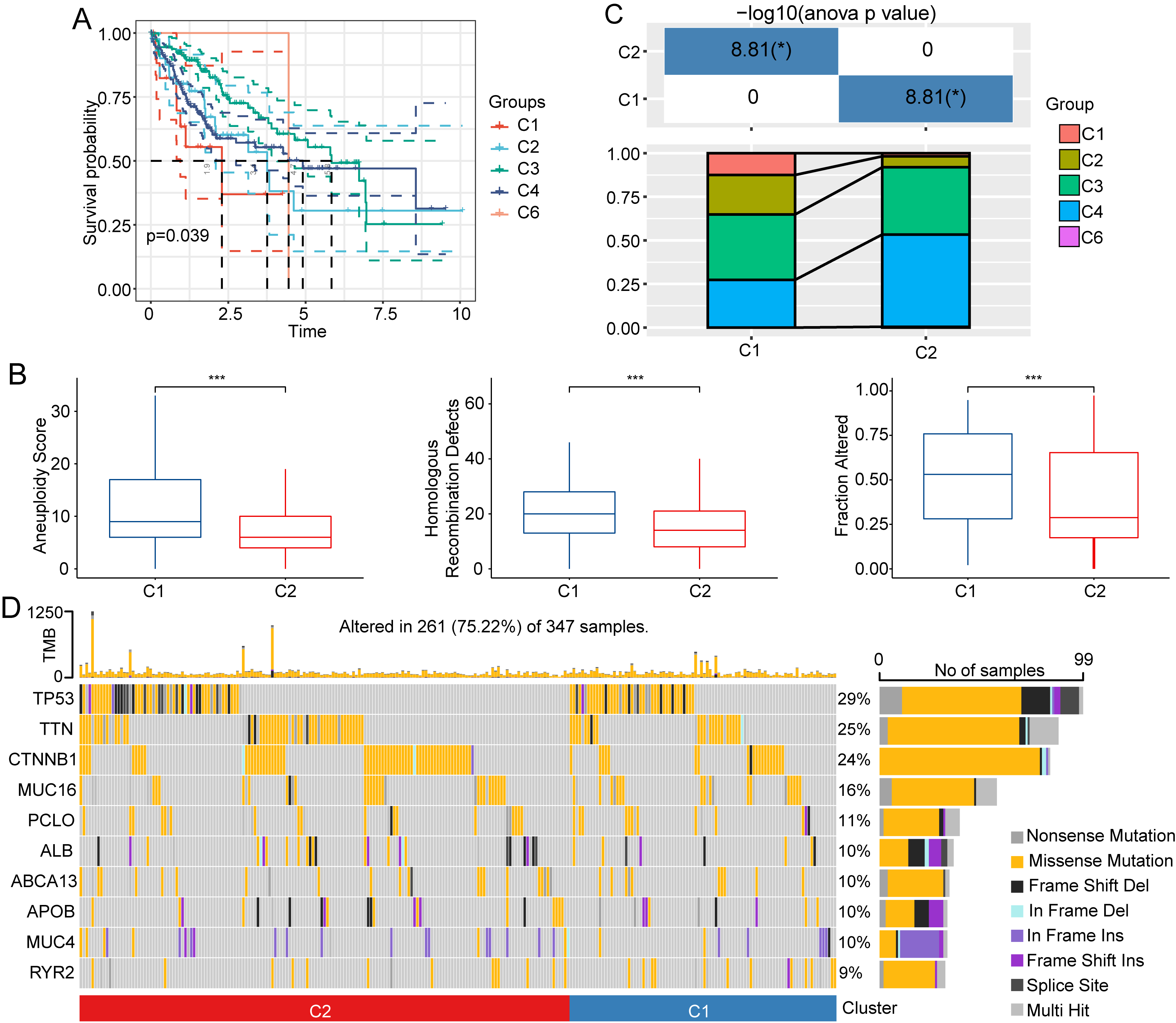

Supplement: Supplementary file 2 — Additional file 2: Figure S2. Genomic alterations of metabolic subtypes in the TCGA-LIHC cohort. [file 12935_2022_2730_MOESM2_ESM.tif]

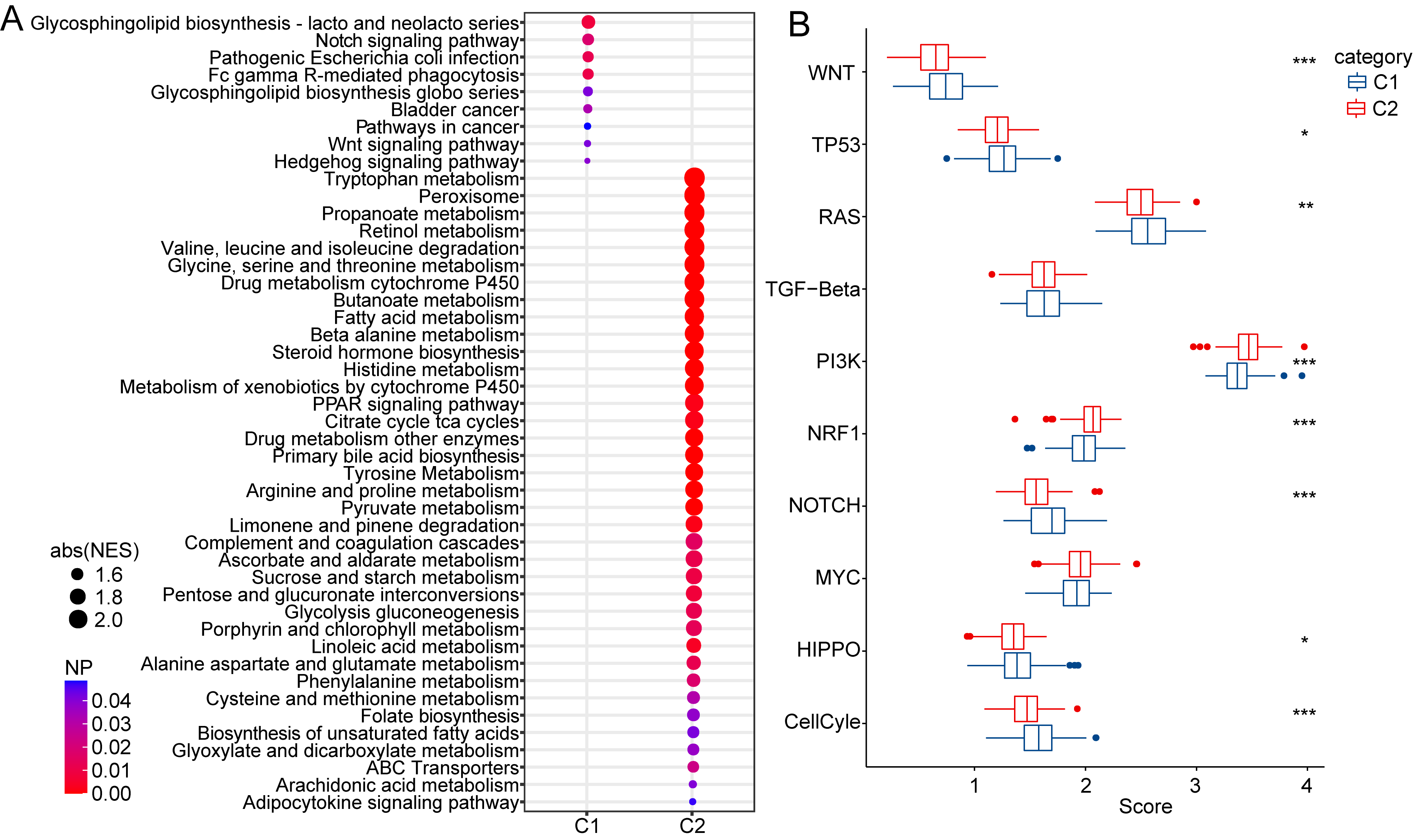

Supplement: Supplementary file 3 — Additional file 3: Figure S3. Enrichment analysis of metabolic subtypes. [file 12935_2022_2730_MOESM3_ESM.tif]

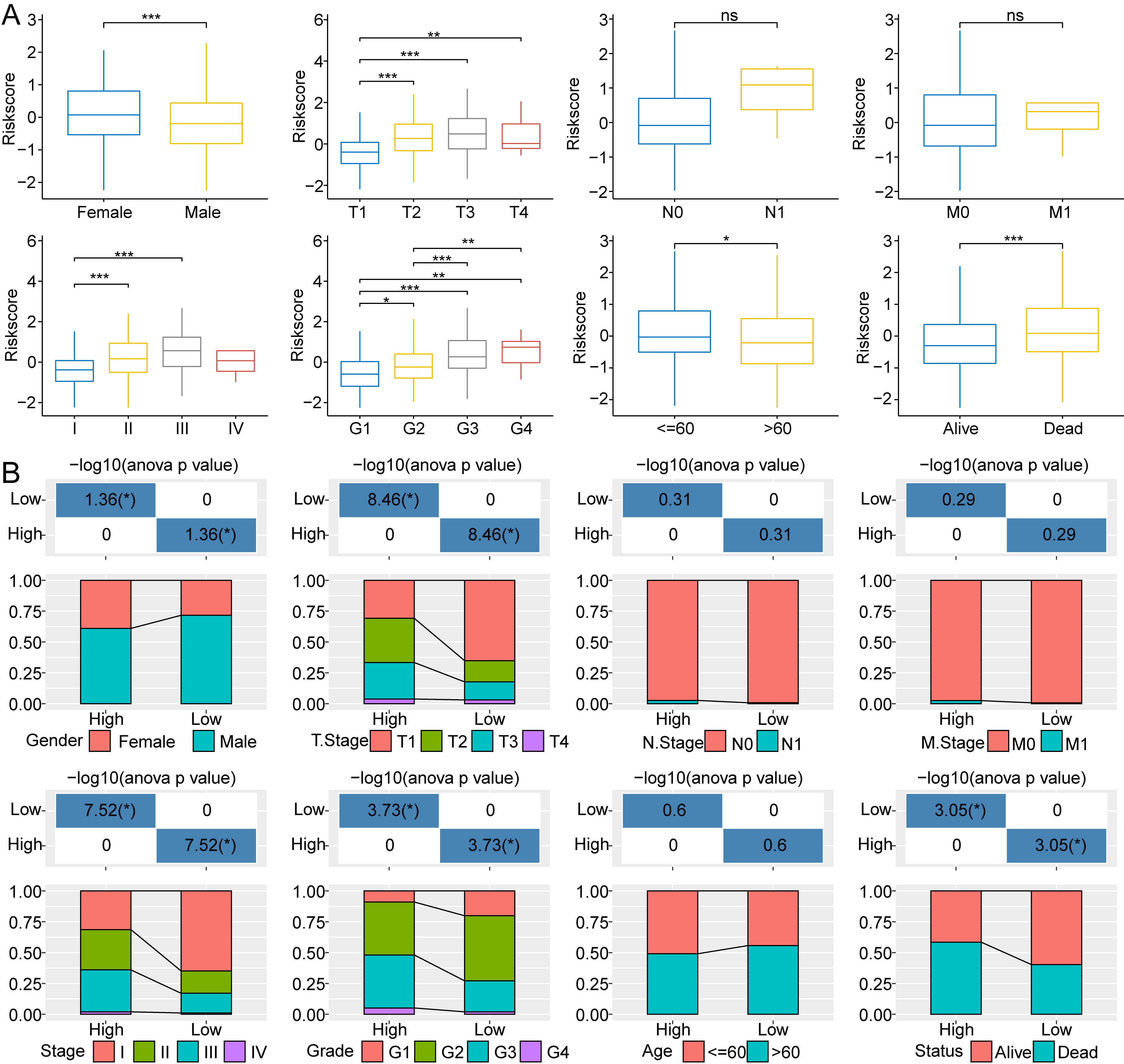

Supplement: Supplementary file 4 — Additional file 4: Figure S4. Characters in different clinicopathological features. [file 12935_2022_2730_MOESM4_ESM.tif]
