# Supplementary material for: Prediction of hepatocellular carcinoma prognosis and immunotherapeutic effects based on tryptophan metabolism-related genes
Source: Cancer Cell Int. 2022 Oct 10;22:308. doi: 10.1186/s12935-022-02730-8 (PMC9552452; doi:10.1186/s12935-022-02730-8)
Supplement: Supplementary file 5 — Additional file 5: Table S1. The information of primers sequences for qRT-PCR assay. [file 12935_2022_2730_MOESM5_ESM.docx]

Supplementary Table 1. The information of primers sequences for qRT-PCR assay.

| Primer name | Sequence (5'-3') |
| --- | --- |
| GAPDH-R | GTCATGAGTCCTTCCACGATACC |
| GAPDH-F | GGAGTCCACTGGCGTCTTCA |
| TPH1-R | AAGCCAGCACCAAAGACTCTTAGC |
| TPH1-F | CAGAGCCAGATACCTGCCATGAAC |
